# Identification of Zirconia Particle Uptake in Human Osteoblasts by ToF-SIMS Analysis and Particle-Size Effects on Cell Metabolism

**DOI:** 10.3390/nano12234272

**Published:** 2022-12-01

**Authors:** Alexander Welle, Kerstin Rabel, Matthias Schwotzer, Ralf Joachim Kohal, Thorsten Steinberg, Brigitte Altmann

**Affiliations:** 1 Karlsruhe Nano Micro Facility (KNMF), Karlsruhe Institute of Technology (KIT), 76344 Eggenstein-Leopoldshafen, Germany; 2 Institute of Functional Interfaces, Karlsruhe Institute of Technology (KIT), 76344 Eggenstein-Leopoldshafen, Germany; 3 Department of Prosthetic Dentistry, Medical Center-University of Freiburg, Faculty of Medicine, University of Freiburg, 79106 Freiburg im Breisgau, Germany; 4 Department of Oral Biotechnology, Center for Dental Medicine, Medical Center-University of Freiburg, Faculty of Medicine, University of Freiburg, 79106 Freiburg im Breisgau, Germany; 5 G.E.R.N. Research Center for Tissue Replacement, Regeneration & Neogenesis, Department of Prosthetic Dentistry, Medical Center-University of Freiburg, Faculty of Medicine, University of Freiburg, 79085 Freiburg im Breisgau, Germany

**Keywords:** zirconia, nano/microparticles, Time-of-Flight Secondary Ion Mass Spectrometry, human alveolar bone, alamarBlue assay

## Abstract

As the use of zirconia-based nano-ceramics is rising in dentistry, the examination of possible biological effects caused by released nanoparticles on oral target tissues, such as bone, is gaining importance. The aim of this investigation was to identify a possible internalization of differently sized zirconia nanoparticles (ZrNP) into human osteoblasts applying Time-of-Flight Secondary Ion Mass Spectrometry (ToF-SIMS), and to examine whether ZrNP exposure affected the metabolic activity of the cells. Since ToF-SIMS has a low probing depth (about 5 nm), visualizing the ZrNP required the controlled erosion of the sample by oxygen bombardment. This procedure removed organic matter, uncovering the internalized ZrNP and leaving the hard particles practically unaffected. It was demonstrated that osteoblasts internalized ZrNP within 24 h in a size-dependent manner. Regarding the cellular metabolic activity, metabolization of alamarBlue by osteoblasts revealed a size- and time-dependent unfavorable effect of ZrNP, with the smallest ZrNP exerting the most pronounced effect. These findings point to different uptake efficiencies of the differently sized ZrNP by human osteoblasts. Furthermore, it was proven that ToF-SIMS is a powerful technique for the detection of zirconia-based nano/microparticles that can be applied for the cell-based validation of clinically relevant materials at the nano/micro scale.

## 1. Introduction

In dental medicine, zirconia-based nanomaterials are gaining importance due to their improved mechanical properties (reviewed in [1,2]). Their application areas concern dental restoration and implant materials [2,3]. The use of zirconia-based nanomaterials makes the examination of possible effects caused by released nanoparticles on oral target tissues important. In this context, numerous studies from the fields of orthopedics and oral implantology point to a nano/microparticle-mediated tissue inflammation with subsequent bone loss [4,5]. In case of oral implants, an accumulation of nano/microparticles has been observed in the peri-implant hard and soft tissues around zirconia oral implants [5,6,7,8]. The assumed mechanisms of particle release are the formation of debris during implant insertion and/or the aging process of zirconia ceramics during their functional period [8]. One important factor is the particle size, which is in addition to the shape, material composition and physicochemical surface properties pivotal for the cell–particle interaction, namely the cellular internalization and cytotoxicity of the particles [9,10,11,12]. As the majority of studies focused on titanium-, gold-, silver- and polymer-based materials, there is, however, only little information on the cell response to zirconia-based nanoparticles (ZrNP). Preliminary data of the cell–ZrNP interactions suggest particle-induced unfavorable effects on cell viability, proliferation and mitochondrial function in different cell types, including tumor cells [13], skin keratinocytes [14], osteoblast-like cells [15,16,17] and primary mouse osteoblasts [18]. Since it has been further demonstrated that the uptake and cytotoxicity of particles strongly depends from the cell type (summarized in [19]), investigations using oral implant-relevant target cells, namely bone-forming osteoblasts from human alveolar bone, may help to reveal the role of released zirconia-based particles during peri-implant bone regeneration and/or inflammation.

A relevant obstacle in the investigation of the cell–ZrNP interaction is the fact that the internalization of ZrNP into human cells cannot be proven by standard optical detection methods, like ordinary light microscopy or fluorescence-based microscopy, as zirconia particles are colorless and non-fluorescent. In order to detect label-free NP uptake in cells and/or tissues, innovative developments in optical and spectral technologies, including confocal reflectance microscopy, darkfield, interferometric and plasmonic imaging, have proved to be suitable to visualize non-labeled particles at the nanoscale (summarized in Priest et al. [20] and Friedrich et al. [21]). However, since most of these systems are based on light reflection and scattering, they do not provide information on the chemical specificity of the signals. i.e., the detected signals may also originate from other structures in the samples (e.g., dust or cell/tissue components) with a similar high refractive index as the particles under study [21]. The shortcomings associated with optical microcopy can be overcome by using Time-of-Flight Secondary Ion Mass Spectrometry (ToF-SIMS). Due to its high sensitivity and spatial resolution, imaging capabilities and versatility, ToF-SIMS is a powerful technology to analyze the interactions of small organic molecules, both natural [22,23] as well as synthetic, and inorganic particles [24,25,26] with cells and tissues [27,28]. Being an ultra-high vacuum method, sample preparation includes similar steps to electron microscopy, namely freezing [29,30] or drying [31], however, most target compounds do not have to be labelled and sample coating with gold or palladium is not required.

Against this background, the aim of the present investigation was to establish ToF-SIMS analysis as a tool to detect the possible internalization of differently-sized zirconia nano/microparticles by alveolar bone osteoblasts derived from oral target tissue and to examine whether zirconia particle exposure affected the metabolic activity of the cells. We therefore used zirconia nanoparticles with different sizes and focused on the evaluation of putative long-term effects, i.e., up to 14 days, on the metabolic activity of osteoblasts after a short particle exposure time of 4 h and 24 h. The metabolic activity was determined by the reduction of the redox-sensitive reporter dye resazurin to fluorescent resorufin in the mitochondrial respiratory chain.

## 2. Materials and Methods

### 2.1. Preparation of Particle Solutions

The zirconia-based particles were purchased as nanopowders from Tosoh Corporation, Tokyo, Japan and Daiichi Kigenso Kagaku Kogyo Co., Ltd., Osaka, Japan. The nominal average sizes of the particles were—according to the manufacturer’s information—as follows: PH < 30 nm, TZ-0 74.8 nm, UEP 460 nm and TZ-3YSE 600 nm (see also Table 1). Prior to use in cell culture, the particles were sterilized by mixing 1 g nanopowder with 70% v/v ethanol in water. This particle–ethanol suspension was then dried at 60 °C until the material was powdery again. For the cell culture experiments, a sterile stock solution of 1 g/mL in cell culture medium was prepared, which was further diluted to the final working concentration of 10 µg/mL. To avoid the formation of particle-aggregates, the stock solution was first treated with ultrasound in an ultrasound bath for 10 min before preparing the diluted working solution.

### 2.2. Isolation and Cultivation of Primary Human Osteoblsts

Primary human osteoblasts were obtained from alveolar bone explants that were harvested during implant site preparation from two healthy male donors (42 and 55 years). The collection and usage of the primary osteoblasts for scientific purposes was approved by the Ethics Committee of the Albert-Ludwigs-University, Freiburg, Germany (vote Nr. 411/08). Informed consent was given by the patients. The bone explants were cleaned in phosphate-buffered saline (PBS) to remove residues of blood and soft and/or bone marrow tissue, sterilized in an iodide solution, subsequently washed with PBS and transferred to Petri dishes. Osteoblasts were then obtained by cell outgrowth from the bone explants. The bone explants, and later the isolated cells, were cultured in Dulbecco’s Modified Eagle’s Medium (Life Technologies, Darmstadt, Germany) supplemented with 1% (w/v) glutamine (Life Technologies, Darmstadt, Germany), 10% (w/v) fetal calf serum (Biochrom AG, Berlin, Germany) and 0.2% (w/v) kanamycin (Sigma-Aldrich, Taufkirchen, Germany). The cells were maintained in a humidified 37 °C incubator with 5% CO_2_. All experiments were carried out with osteoblasts of passage 6.

### 2.3. Scanning Electron Microscopy (SEM)

An Environmental Scanning Electron Microscope (ESEM) type XL 30 from Philips equipped with a field emission gun was used for the electron microscopic investigations. Prior to the investigations, the samples were conductively coated with platinum (Pt, ntracex. 3 nm).

### 2.4. Secondary Ion Mass Spectrometry (SIMS) Analysis

For the detection of particle internalization into osteoblasts by SIMS, 1.3 × 10^4^ cells per cm^2^ were cultured on sterile silicon wafers (duplicates) which were coated with 1 µg/mL human fibronectin solution (Sigma-Aldrich, Taufkirchen, Germany). After 24 h preculture on the silicon wafers, the cells were incubated for 4 h or 24 h with the different zirconia particles, washed with phosphate buffered saline (PBS) and fixed with 4% formaldehyde in PBS for 20 min at room temperature. The applied incubation times were chosen based on preliminary experiments with fluorescent polystyrene-based nanoparticles (Polysciences Europe GmbH, Eppelheim, Germany) with 50 nm and 500 nm size indicating an uptake of 50 nm nanoparticles already after 4 h incubation (see also Figure S1). Samples were then dehydrated in ascending ethanol series (ranging from 30% to 100% ethanol, three times each for 20 min at room temperature) and critical point dried (CPD030 Critical Point Dryer; Bal-Tec AG, Balzers, Liechtenstein).

After fixation and drying, the samples were analyzed by SIMS performed on a TOF.SIMS5 instrument (ION-TOF GmbH, Münster, Germany) equipped with a Bi-cluster primary ion source and a reflectron type analyzer. Ultra high vacuum (UHV) base pressure was <9 × 10^−9^ mbar. The final experimental protocol consisted of 4 steps: In Step 1, three spots on each sample were analyzed within a static SIMS mode having very high sensitivity and about 5 nm probing depth. Therefore, a bunched beam of 25 keV Bi^+^ was used for primary beam scanning over 500 × 500 µm^2^ fields of view (128 × 128 pixel, 50 scans, dose density 3 × 10^11^ 1/cm^2^, positive secondary ion polarity). The short pulse length of 1.1 ns allowed for high mass resolution. Spectra were calibrated on the omnipresent C^+^, CH^+^, CH_2_^+^, CH_3_^+^, and Si^+^ peaks. Next (step 2), the samples were eroded by applying a 2 keV oxygen beam while simultaneously recording secondary ions. Therefore, in addition to the Bi beam, a 2 keV oxygen beam was scanned across a concentric field of 700 × 700 µm^2^ (dose density 7.5–8 × 10^16^ 1/cm^2^). This beam effectively degrades and erodes organic matter and our preliminary experiments showed low damage to the hard zirconia particles. Following the erosion run another static SIMS run was performed (step 3) showing the location of the exposed zirconia particles. Finally (step 4), a high lateral resolution scan of the eroded area was performed with a non-bunched primary ion beam (burst alignment mode, 500 × 500 µm^2^ fields of view, 512 × 512 pixel, 150 scans). Original data with high lateral resolution can be found at DOI: 10.35097/728 (both donors, all ZrNP and negative control, 4 resp. 24 h incubation), original data for all steps of this procedure are given in \preparation flow.zip (for TZ-0 particles). To develop and demonstrate this analysis process a preliminary experiment was performed differently allowing for recording of high resolution 3D datasets; see repository Radar4KIT, DOI: 10.35097/728: \high depth resol\24h_donorA_PH 1571_4.zip and 24h_donorA_PH 1571_5.zip (for PH particles). If not noted otherwise, for imaging a red/green/blue overlay of the high lateral resolution images after erosion is presented with the sum of Na, K, Ca and CaF (showing the footprints of the cells) in the red channel, the sum of zirconium isotopes and ZrO^+^ isotopes in the green channel and the underlying Si^+^ in the blue channel.

### 2.5. Analysis of the Metabolic Activity of Osteoblasts

The osteoblast’s mitochondrial metabolic activity was measured by using the alamarBlue assay (Bio-Rad, Hercules, CA, USA). The redox indicator alamarBlue (AB) is composed of non-fluorescent resazurin that is reduced/metabolized in the mitochondrial respiratory chain of living cells to fluorescent resorufin. Subsequently, the amount of resorufin in the cell culture medium can be quantified by fluorimetry as an indicator of the cell´s mitochondrial metabolic activity. Hence, the nanoparticle solution was removed after 4 h or 24 h of incubation and replaced by a cell culture medium supplemented with 10% (w/v) AB. After 3 h of incubation with 10% AB containing medium, the supernatant was collected and a standard culture medium was added for further cell culture. After 7 and 14 days, the medium was removed and as described above replaced by AB medium for 3 h. This procedure has proved to be appropriate for primary human osteoblasts in former work [32]. The reduced AB, i.e., resorufin, was quantified in the supernatant by measuring the fluorescence at 590 nm with a microplate reader (Infinite m200, Tecan, Männedorf, Germany). The percentage of AB metabolization in the samples was calculated as described by the manufacturer by using a 100% reduced AB control as reference, which was produced according to the manufacturer’s instructions by autoclaving a sample containing culture medium with 10% (w/v) AB reagent for 15 min.

### 2.6. Statistical Analysis

The cell culture experiments for the alamarBlue assay were performed in five independent experiments with triplicates. The Shapiro–Wilk test was used to test if the data were normally distributed. Since the data were normally distributed, we used the Paired t-test to identify significant differences between the test groups. The statistical data analysis and visualization was performed with OriginPro 2021b.

## 3. Results and Discussion

### 3.1. Scanning Electron Microscopy

**Figure 1:**
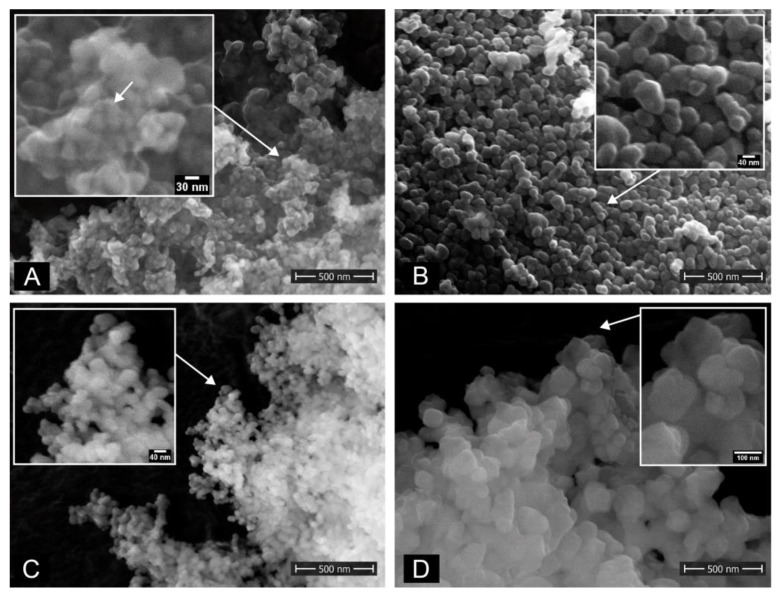
SEM images of plain nanoparticles as received. (**A**) PH particles, (**B**) TZ-0 particles, (**C**) UEP particles, (**D**) TZ-3YSE particles. The arrow in the insert of Figure 1A indicates a possible coating of the particles.

In order to visualize the actual shapes and dimensions of the zirconia particles (ZrNP) under study, scanning electron micrographs were recorded, see Figure 1. For particle samples PH (Figure 1A) and TZ-0 (Figure 1B) manufacturers’ information were consistent with our findings. UEP (Figure 1C) and TZ-3YSE (Figure 1D) consisted of aggregates of smaller individual particles. Mean particle size data provided by the manufacturers for UEP and TZ-3YSE therefore seemed rather to reflect aggregate sizes of the powder samples. As shown, PH particles had the smallest grain size with <40 nm (Figure 1A), TZ-0 (Figure 1B) and UEP (Figure 1C) had similar grain sizes ranging from 40 to 80 nm, and TZ-3YSE (Figure 1D) were found to be considerably larger with >100 nm than the other used particles. Furthermore, none of the used ZrNP showed a porous structure.

### 3.2. Establishment of ToF-SIMS for the Detection of Intracellular ZrNP in Human Osteoblasts

**Figure 2:**
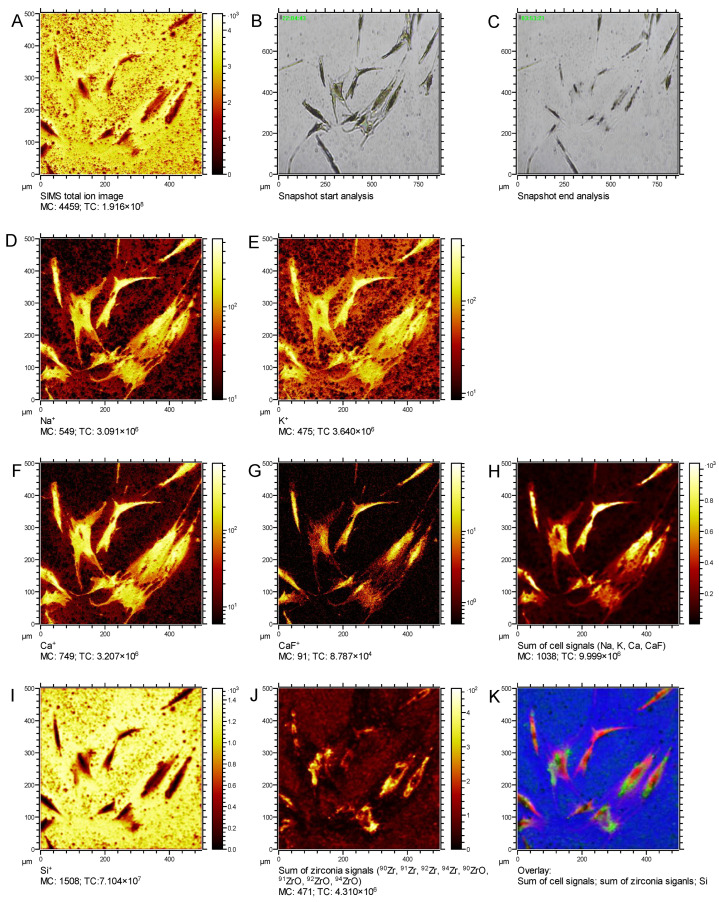
Depth-integrated SIMS-images of an erosion experiment performed with cells incubated with PH-ZrNP for 24 h. The image panel includes a total secondary ion image (**A**), optical images before (**B**) and after erosion (**C**), images of the cellular components sodium (**D**), potassium (**E**), calcium (**F**) and calcium fluoride (**G**) and of the sum of these cellular signals (**H**), a silicon (**I**) and zirconia (**J**) image. (**K**) RGB overlay of the cellular (**H**), zirconia (**J**) and substrate signals (**I**) signals. Zirconia particles are visualized in green, the cell bodies are red and the silicon substrate is given in blue.

**Figure 3:**
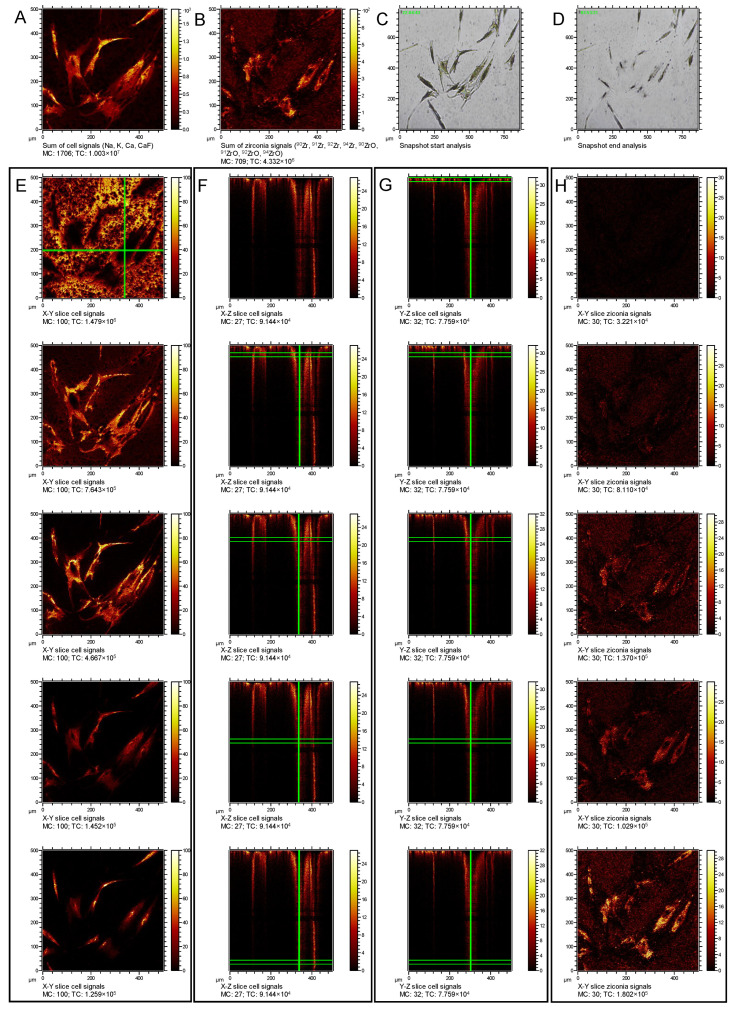
Depth integrated combined signals from cellular components (**A**) and ZrNP (**B**), optical micrographs before (**C**,**D**) after erosion. Cross sectional cellular (**E**–**G**) and zirconia (**H**) signals. First row (**E**,**H**), show the signals at the beginning of the erosion. (**E**) X–Y slice of cellular signals; X–Z (**F**) and Y–Z (**G**) slices cut thereof at the green lines displayed in the images. (**H**) X–Y slice of zirconia signals at the beginning of the erosion as indicated by green lines in this column. The following rows are again showing the same signals with gradually deeper layers for the cellular signals and zirconia signals.

In order to evaluate the suitability of the ToF-SIMS method to identify a putative uptake of ZrNP by osteoblasts in vitro, we performed a pilot erosion experiment with cells exposed to PH-ZrNP for 24 h. A 3D dataset with high resolution was recorded from cells incubated with PH-ZrNP for 24 h. All original files from the dataset shown below and another example are available from a repository (DOI: 10.35097/728, \high depth resol\24h_donorA_PH 1571_5.zip). The procedure for method development differed slightly from the conventional procedure since instead of the 2 keV oxygen beam, for this experiment a 1 keV oxygen beam was used to erode the sample slower, and data recording was performed with a non-bunched Bi primary beam with 256 × 256 pixel (x/y) in a non-interlaced sequence with 301 z-layers. This experiment required 6 h of recording time, yielded large raw data files (>3 GB) and possessed technical challenges since minute thermal drifts in the spectrometer had to be avoided or corrected. The data of this experiment is presented as depth-integrated images (Figure 2) and as calculated cross sections through the 3D data space (Figure 3). Note that the z-axis is not to scale and the true topography of the sample is not taken into account.

Figure 2 shows depth-integrated X–Y images, (A) total secondary ions, and video snapshots at start (B) and end (C) of the measurement, depth integrated secondary ion images of the cellular components Na^+^ (I K^+^ (E), Ca^+^ (F), CaF^+^ (G) and the sum of these elements (H)). Secondary ion images of the silicon substrate and zirconia distribution are shown in Figure 2I,J, respectively. Furthermore, the overlay of the cellular components, silicon and the zirconia signals (K) enable the precise localization of the zirconia particles in and/or around the cells.

The localization of the zirconia particles and their internalization by the cells are illustrated in Figure 3. The first row depicts depth integrated signals of cellular components (A) and depth integrated signals indicating the ZrNP (B), as well as optical images of the start (C) and end (D) point. Below, Figure 3E–H show calculated cross sections through the 3D data set. Figure 3E depicts thereby the X–Y slice of cellular signals in the topmost layer of the specimen at the onset of the erosion experiment. Figure 3F,G depict cross sections of these signals along the other two axes, whereas Figure 3H shows the lateral distribution of the ZrNP within the topmost layer of the sample. For the images below, the corresponding X–Y cross sections of cellular signals and ZrNP signals are provided progressing deeper into the sample as indicated by the green lines. As shown in Figure 3, images of the cellular components (E and below) and zirconia (H and below) revealed changes of the signal intensities during the erosion of the specimen which were characterized by an overall decline of the cell-associated signals (E and below) and a simultaneous rise of the zirconia signals (H and below). At first, with only slight oxygen bombardment, Na^+^, K^+^, Ca^+^ and CaF^+^ signals originated mainly from extracellular deposits and no zirconia particles were detected (compare in Figure 3E,H; first row). When progressing through the erosion process (Figure 3E,H, subsequent rows) the extracellular Na^+^, K^+^, Ca^+^ and CaF^+^ signals were dropping since the thin layer of extracellular matrix was already fully eroded (Si^+^, not shown, became more prominent). In the time course of the erosion the extracellular signals decreased from the cell’s circumference as these thinner parts and filopodia became more and more removed by the oxygen bombardment. Simultaneously, more and more zirconia particles became uncovered following the shape of the cells but leaving out the position of the cell´s nuclei. These data show that a controlled erosion of the fixed cells is possible, and that thinning down the adherent cells gradually revealed intracellular ZrNP, which were distinguishable from extracellular particles on the substrate in between the cells.

### 3.3. Identification of Size- and Time-Dependent ZrNP Internalization into Osteoblasts by ToF-SIMS

After having established the experimental protocol to erode the specimen and record high lateral resolution images of ZrNP by SIMS, a series of runs with two different incubation times and the differently-sized ZrNP were performed, including four individual SIMS analyses steps as described in Section 2.4. At initial static SIMS analysis (step 1), samples showed no or very weak Zr^+^ and ZrO^+^ signals. This was due to the fact that even non-internalized particles become rapidly covered by adsorbed proteins from the cell culture media [33,34,35,36,37]. This adsorbate layer effectively shielded zirconia particles from being probed by SIMS. To expose zirconia particles internalized or covered in proteinaceous adsorbates, small and aggressive sputter ions, like O_2_^+^, were especially useful since organic molecules yielding a plethora of signals in the mass spectrum were degraded and removed rapidly. Although some prominent markers for proteinaceous materials, e.g., the CH_4_N^+^ fragment, were also degraded, intracellular sodium, potassium and calcium provided a clear contrast of the cell’s footprint to the surrounding silicon from the wafers used for cell culture. Compared to Veith et al. [24] regarding a comparison between different sputter beams, O_2_^+^ versus Ar_1000_^+^, the latter allowed for recording of numerous amino acid-derived secondary ions like C_3_H_8_N, C_4h8_N and C_6_H_12_N. Zirconia shows due to its hardness and a much slower erosion and the ionization yield was enhanced by oxygen implantation. Both effects became important for high lateral resolution imaging (step 4). The used analysis mode offered only limited sensitivity as compared to the initial static SIMS mode with bunched primary beam, and only nominal mass resolution. Oxygen sputtered samples lost the majority of signals from organic compounds in the high mass range, therefore Zr^+^ together with ZrO^+^ signals became prominent and unambiguous. Both signal multiplets showed the expected isotope distribution of zirconium.

**Figure 4:**
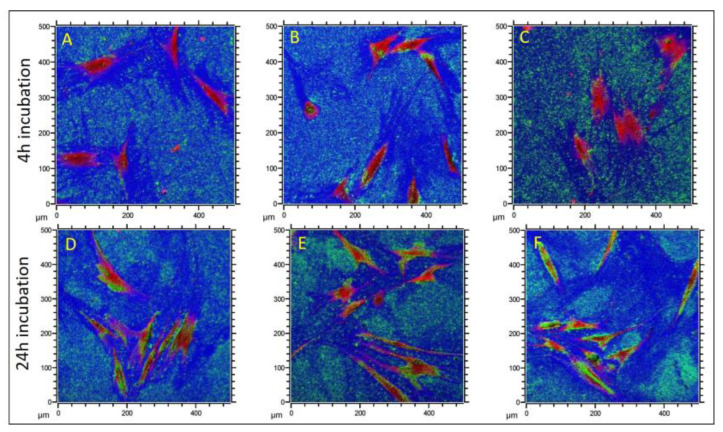
Post erosion, high lateral resolution ToF-SIMS images derived from three positions showing cells incubated with TZ-0 particles for 4 h (**A**–**C**) and 24 h (**D**–**F**). Sum of Na^+^, K^+^, Ca^+^ and CaF^+^ signals (showing the footprints of the cells) in the red channel. Sum of zirconium isotopes and ZrO^+^ isotopes in green channel, and the underlying Si in the blue channel.

Figure 4 shows representative overlay images of cells, labeled in red, which were incubated with TZ-0 particles, labeled in green, for 4 h (A–C) and 24 h (D–F), and the silicon substrate in blue. As displayed in Figure 4A–C the ZrNP have sedimented down after 4 h incubation time; however, cells avoided the particles instead of internalizing them. With a prolonged incubation, i.e., 24 h, the osteoblasts accumulated the TZ-0 particles as shown in Figure 4D–F. The ZrNP were enriched in the cytoplasm and only the central area of the cells, where the gap between the cell nucleus and the outer membrane was narrow, remained devoid of zirconia particles. The results of the SIMS analysis for all ZrNP and incubation times are summarized in Table 2 and demonstrate that PH and TZ-0 particles were clearly internalized by human osteoblasts after 24 h exposure. The uptake of UEP was in part detectable and the internalization of the biggest particles TZ-3YSE was less prominent than for the smaller ZrNP. This indicates that the particle size was decisive for the time course of particle uptake, with an apparent slower internalization of particles with increasing particle size. This observation was backed up by our preliminary experiments with green fluorescent nanoparticles, demonstrating an uptake of 50 nm but not of 500 nm particles (see also Figure S1). These findings are furthermore in accordance with other reports using polystyrene [38], gold [39] and silica [40] nanoparticles. With respect to the presented experimental protocol, it should be mentioned here that the detailed process description and the availability of analysis parameters and raw data via our datasets in the repository Radar4KIT (DOI:10.35097/728) is intended to facilitate future experiments based on this powerful technology.

### 3.4. Effects of ZrNP on the Metabolic Activity of Osteoblasts

Since SIMS analysis revealed that ZrNP internalization was detectable after 24 h incubation, we were next interested in whether the ZrNP uptake affected the metabolic activity of the osteoblasts. Therefore, we examined the metabolization of the redox-sensitive reporter dye resazurin (alamarBlue), which is reduced in the mitochondrial respiration and thus provides information on the metabolic activity of the cells. Again, the osteoblasts were exposed to ZrNP for 4 h and 24 h, and the alamarBlue (AB) reporter dye product resorufin was quantified in the supernatant directly after the respective incubation time day 0 (immediately after ZrNP incubation), or after 7- and 14-days culture.

**Figure 5:**
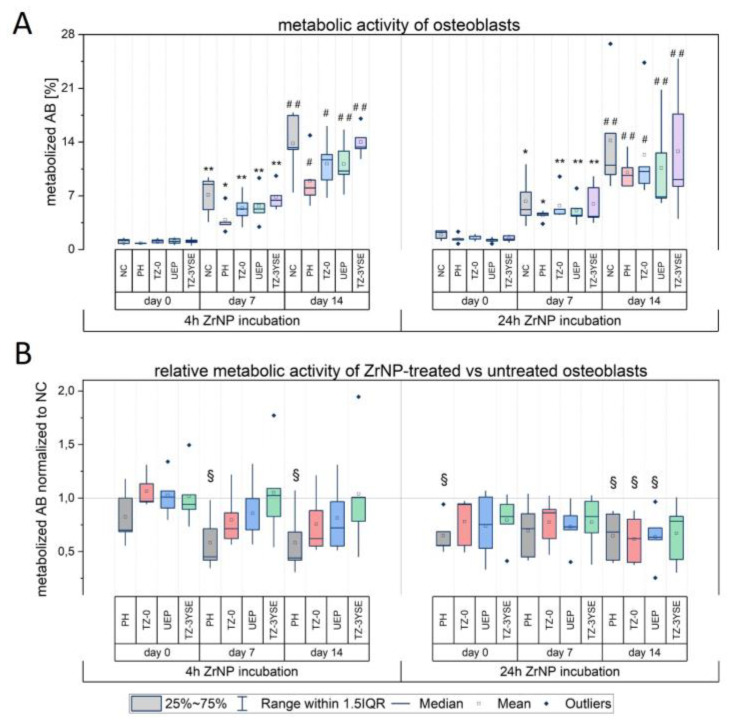
(**A**) Metabolic activity of osteoblasts determined by the amount of metabolized alamarBlue (AB) reporter dye product in the supernatant at day 0, 7 and 14 of culture. Day 0 indicates the AB metabolization directly after incubation. The AB metabolization rate is given as percentage of a 100% reduced medium control according to the manufacturer. (**B**) Relative metabolic activity of ZrNP treated and untreated cells. A +ZrNP/-ZrNP ratio of 1 means no effect, i.e., AB metabolization in ZrNP-treated cells is equal to untreated cells. * *p* < 0.05 and ** *p* < 0.01 for comparison of AB metabolization at day 7 versus day 0; ^#^ *p* < 0.05 and ^##^ *p* < 0.01 for comparison of AB metabolization at day 14 versus day 7; ^§^ *p* < 0.05 for comparison of AB metabolization between ZrNP-treated and untreated cells (NC); Paired *t*-test, n = 5.

The results shown in Figure 5A demonstrate that the percentage of metabolized AB increased, irrespective of the ZrNP incubation time, until day 14 in ZrNP-treated and untreated osteoblasts (negative control; NC). The rise in the percentage of metabolized AB over culture time thereby indicates that the osteoblasts were viable and proliferated after ZrNP exposure. This demonstrates that applied ZrNP concentration and the chosen particle incubation times, i.e., 4 h and 24 h, in our approach were subtoxic for the osteoblasts.

In order to assess the effect of ZrNP exposure on the metabolic activity of the cells, we next compared the AB metabolization rates in ZrNP-treated versus untreated osteoblasts. In Figure 5B, the effect of ZrNP treatment is given by the +ZrNP/-ZrNP ratio of measured metabolized AB product in the supernatant and describes the fold change of the AB metabolization in ZrNP-treated versus corresponding untreated cell cultures. A ratio of 1 means no effect, i.e., AB metabolization in ZrNP-treated cells is equal to untreated cells. As presented in Figure 5B, 4 h ZrNP incubation with the smallest particles PH caused a lower metabolic activity of osteoblasts after 7 days (*p* = 0.02341) and 14 days (*p* = 0.03746) culture post-exposure when compared with untreated controls (indicated as reference line at 1 in Figure 5B). A similar trend was observable in cell cultures incubated for 24 h with PH with significant lower AB reduction values already measurable directly after incubation (d0, *p* = 0.01154). Interestingly, at day 14 post-exposure AB metabolization was not only decreased in cells incubated for 24 h with PH-ZrNP (*p* = 0.02584), but also in osteoblasts treated with TZ-0 (*p* = 0.01992) and UEP (*p* = 0.03391) particles. These data demonstrate that among the tested ZrNP, the smallest particles, namely PH, had the most considerable effect on the cell’s metabolic activity, as lower values with matched controls were detectable after short as well as prolonged exposure and culture time. Particles with the next larger sizes, namely TZ-0 and UEP, unfolded their effect on cell metabolism only after 24 h ZrNP incubation time and cell treatment with the largest particles TZ-3YSE had no significant effect. Hence, our findings reveal a size- and time-dependent unfavorable effect of ZrNP on the metabolic activity of human osteoblasts, with smaller particles exerting a faster effect on cellular metabolism. It should be further highlighted that the lower AB metabolization rate at days 7 and 14 in cells incubated for 4 h with PH-ZrNP points to a particle uptake after short-term exposure, even though intracellular ZrNP were not detected by SIMS at this time point. A possible reason might be that SIMS represents a qualitative analysis method that visualizes only a part of the whole sample, whereas the AB assay reflects the cellular metabolism of the whole cell population. Hence, it is conceivable that the internalization of PH-ZrNP was already initiated at an early stage of incubation. The lower metabolic activity of osteoblasts after ZrNP exposure is furthermore in line with other studies reporting adverse effects of ZrNP on the structural and functional integrity of mitochondria [13,14] and a size-dependent inhibition of metabolic enzymes by gold nanoparticles [17].

Taken together, the results from the AB assay and SIMS analysis demonstrate a particle internalization of PH, TZ-0 and UEP versus a less prominent uptake of the bigger TZ-3YSE particles and thus point to a different uptake efficiency of ZrNP in human osteoblast cultures. Since different entry pathways of nano/microparticles have been described in various cell types, including endocytosis and phagocytosis (summarized in [19,41]), the underlying mechanism of cellular ZrNP internalization in human osteoblasts deserves further study. Since the SIMS technology enables the identification and localization of nanoparticles in vitro, i.e., intra- and/or extracellular space, the established protocol presented in the current work provides a useful supplement for the systematic investigation on the cellular particle uptake and particle-associated effects on cell behavior.

## 4. Conclusions

The identification of nano/microparticles in peri-implant hard and soft tissues around zirconia oral implants raised the question about the biological effect of zirconia-based particles on oral target cells such as bone-forming osteoblasts. Therefore, we focused in the present study on the examination of the putative internalization of zirconia-based nanoparticles by human alveolar bone osteoblasts and on the impact of these particles on the metabolic activity of the cells. In order to identify particle internalization by osteoblasts, we successfully established Time-of-Flight Secondary Ion Mass Spectrometry (ToF-SIMS) as detection technology for intra- and extracellular nano/microparticles. By this, we demonstrated for the first time that primary human osteoblasts are able to internalize ZrNP. As shown, the ZrNP uptake efficiency depended on the size of the ZrNP, as smaller particles < 40 nm were predominantly internalized, and on incubation time. With respect to the biological effect of the particles on osteoblast metabolism, our results further show that particle uptake led to a lower metabolic activity with matched untreated controls. The modulation of the metabolic activity of the cells depended thereby again on the particle size and incubation time. The smallest ZrNP (PH) showed the most considerable effect on cell metabolism. Hence, our findings indicate different uptake kinetics and/or internalization mechanisms of zirconia-based nano/microparticles in human osteoblasts. Based on our results we further conclude that the established ToF-SIMS technology provides in addition to biological assays a powerful tool for the systematic investigation of nano/microparticle-associated effects on cell behavior.

## Figures and Tables

**Table 1:** Nomenclature and composition of zirconia particles used for ToF-SIMS analysis and cell culture experiments. Data provided by manufacturers.

Particle Label	Composition in Addition to ZrO_2_	Manufacturer
PH	No data	Tosoh Corporation, Tokyo, Japan
TZ-0	No data	Tosoh Corporation, Tokyo, Japan
UEP	Fe_2_O_3_: 0.002%, SiO_2_: 0.01%, TiO_2_: 0.001%	Daiichi Kigenso Kagaku Kogyo Co., Ltd., Osaka, Japan
TZ-3YSE	Y_2_O_3_: 5.15%, Al_2_O_3_: 0.25%, SiO_2_: ≤0.02%, Fe_2_O_3_: ≤0.01%, Na_2_O: ≤0.04%	Tosoh Corporation, Tokyo, Japan

**Table 2:** Summarized results of the SIMS analysis of cell cultures incubated with differently-sized ZrNP for 4 h and 24 h. n = 2 (independent experiments with duplicates).

Particle Label	Exposure Time	Particle Internalization
PH	4 h	No
	24 h	Yes
TZ-0	4 h	No
	24 h	Yes
UEP	4 h	No
	24 h	partly
TZ-3YSE	4 h	No
	24 h	possibly

## Data Availability

The data presented in this study are openly available in Radar4KIT at https://doi.org/10.35097/728.

## Supplementary Materials

The following supporting information can be downloaded at: https://www.mdpi.com/article/10.3390/nano12234272/s1, Figure S1. Microscopic analysis of fluorescent nanoparticle uptake by osteoblasts. Bright field and fluorescence images of primary human osteoblasts incubated with 10 µg/mL fluorescent nano-particles (Fluoresbrite, Polyscience Europe GmbH, Eppenheim, Germany) with 50 nm and 500 nm size for 4 h.
