# Supplementary material for: Identification of Zirconia Particle Uptake in Human Osteoblasts by ToF-SIMS Analysis and Particle-Size Effects on Cell Metabolism
Source: Nanomaterials (Basel). 2022 Dec 1;12(23):4272. doi: 10.3390/nano12234272 (PMC9736599; doi:10.3390/nano12234272)
Supplement: Supplementary file 1 [file nanomaterials-12-04272-s001.zip › nanomaterials-2021784-supplementary.pdf]

nanomaterials

Supplementary Materials

Identification of Zirconia Particle Uptake in Human Osteoblasts by ToF-SIMS Analysis and Particle-Size Effects on Cell Metabolism

Alexander Welle [1,2,†], Kerstin Rabel [3,†], Matthias Schwotzer [2], Ralf Joachim Kohal [3], Thorsten Steinberg [4] and Brigitte Altmann [3,5,*]

1 Karlsruhe Nano Micro Facility (KNMF), Karlsruhe Institute of Technology (KIT), 76344 Eggenstein-Leopoldshafen, Germany; alexander.welle@kit.edu
2 Institute of Functional Interfaces, Karlsruhe Institute of Technology (KIT), 76344 Eggenstein-Leopoldshafen, Germany; matthias.schwotzer@kit.edu
3 Department of Prosthetic Dentistry, Medical Center-University of Freiburg, Faculty of Medicine, University of Freiburg, 79106 Freiburg im Breisgau, Germany; kerstin.rabel@uniklinik-freiburg.de (K.R.); ralf.kohal@uniklinik-freiburg.de (R.J.K.)
4 Department of Oral Biotechnology, Center for Dental Medicine, Medical Center -University of Freiburg, Faculty of Medicine, University of Freiburg, 79106 Freiburg im Breisgau, Germany; thorsten.steinberg@uniklinik-freiburg.de
5 G.E.R.N Research Center for Tissue Replacement, Regeneration & Neogenesis, Department of Prosthetic Dentistry, Medical Center -University of Freiburg, Faculty of Medicine, University of Freiburg, 79085 Freiburg im Breisgau, Germany
* Correspondence: brigitte.altmann@uniklinik-freiburg.de
† These authors contributed equally to this work.

Citation: Welle, A.; Rabel, K.; Schwotzer, M.; Kohal, R.J.; Steinberg, T.; Altmann, B. Identification of Zirconia Particle Uptake in Human Osteoblasts by ToF-SIMS Analysis and Particle-Size Effects on Cell Metabolism. *Nanomaterials* **2022**, *12*, 4272. https://doi.org/10.3390/nano12234272

Academic Editor: Wei Chen

Received: 25 October 2022
Accepted: 23 November 2022
Published: 1 December 2022

Publisher's Note: MDPI stays neutral with regard to jurisdictional claims in published maps and institutional affiliations.

Copyright: © 2022 by the authors. Submitted for possible open access publication under the terms and conditions of the Creative Commons Attribution (CC BY) license (https://creativecommons.org/licenses/by/4.0/).

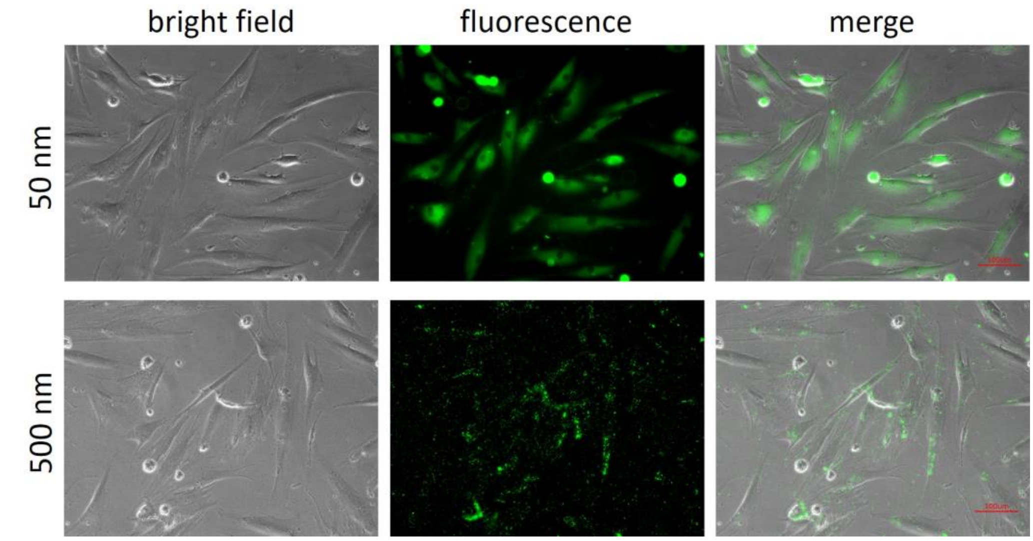

Figure S1. Microscopic analysis of fluorescent nanoparticle uptake by osteoblasts. Bright field and fluorescence images of primary human osteoblasts incubated with 10 µg/ml fluorescent nanoparticles (Fluoresbrite, Polyscience Europe GmbH, Eppenheim, Germany) with 50 nm and 500 nm size for 4 h.
